# Supplementary material for: Evaluating Acylsugars-Mediated Resistance in Tomato against Bemisia tabaci and Transmission of Tomato Yellow Leaf Curl Virus
Source: Insects. 2020 Nov 28;11(12):842. doi: 10.3390/insects11120842 (PMC7760652; doi:10.3390/insects11120842)
Supplement: Supplementary file 1 [file insects-11-00842-s001.pdf]

insects

MDPI

Supplementary Table S1

Table S1. Type III statistics associated with fatty acids percentage from acylsugar lines.

Fatty Acids[a]	Num. DF	Den. DF	F	*p*
i-C4	4	30	44.10	<0.0001
ai-C5	4	30	10.74	<0.0001
i-C5	4	30	25.57	<0.0001
ai-C6	4	30	6.36	0.0012
i-C6	4	30	2.31	0.0871
i-C9	4	30	95.54	<0.0001
i-C10	4	30	32.94	<0.0001
n-C10	4	30	139.33	<0.0001
ai-C11	4	30	238.19	<0.0001
iC-11	4	30	173.09	<0.0001
nC-11	4	30	1.62	0.2013
iC-12			3.51	0.0216
nC-12	4	30	66.21	<0.0001
i-C13			488.23	<0.0001
i-C14	4	30	139.33	<0.0001

* Fatty acids in terms of proportions from five tomato acylsugar tomato accessions are presented.

Supplementary Table S2

Table S2. Type III statistics on whitefly settling assays to assess differences between hosts and leaf surfaces.

**FL47 vs. Cu071026**				
**Effect**	**Numerator Df**	**Denominator DF**	**F value**	***p***
Host	1	11	10.21	0.0085
Leaf surface	1	22	38.15	<0.0001
Host *leaf surface	1	22	123.77	<0.0001
FL47 vs. FA2/CU071026				
Host	1	11	1.13	0.0217
Leaf surface	1	22	31.35	<0.0001
Host *leaf surface	1	22	32.36	<0.0001
FL47 vs. FA7/CU071026				
Host	1	11	7.14	0.0217
Leaf surface	1	22	20.73	0.0002
Host *leaf surface	1	22	24.14	<0.0001
FL47 vs. FA2/FA7/CU071026				
Host	1	11	4.15	0.0665
Leaf surface	1	22	24.08	<0.0001
Host *leaf surface	1	22	24.25	<0.0001
FL47 vs. QTL6/CU071026				
Host	1	11	10.19	0.0086
Leaf surface	1	22	13.01	0.0016
Host *leaf surface	1	22	36.35	<0.0001
Cu071026 vs. FA2/CU071026				
Host	1	11	0.02	0.8865
Leaf surface	1	22	0.10	0.7576
Host *leaf surface	1	22	2.09	0.1619
Cu071026 vs. FA7/CU071026				
Host	1	11	0.00	0.9940
Leaf surface	1	22	8.13	0.0093
Host *leaf surface	1	22	3.47	0.0757
Cu071026 vs. FA2/FA7/CU071026				
Host	1	11	0.03	0.8636
Leaf surface	1	22	6.46	0.0186
Host *leaf surface	1	22	1.57	0.2227
Cu071026 vs. QTL6/CU071026				
Host	1	11	0.14	0.7138
Leaf surface	1	22	1.45	0.2419
Host *leaf surface	1	22	0.55	0.4650

Settling assays were conducted to distinguish differences between non-acylsugar tomato cultivar and acylsugar-producing lines, and also between the benchmark acylsugar line CU071026 and other recently derived acylsugar-producing lines. Host in the table refers to both non-acylsugar cultivar and acylsugar-producing lines. Leaf surface refers to both abaxial (lower) and adaxial (upper) leaf surfaces.

© 2020 by the authors. Licensee MDPI, Basel, Switzerland. This article is an open access article distributed under the terms and conditions of the Creative Commons Attribution (CC BY) license (http://creativecommons.org/licenses/by/4.0/).
